# Telemedicine can be a feasible means of guiding untrained general practitioners to perform point-of-care ultrasound in life-threatening situations: the case of a field hospital during the COVID-19 pandemic

**DOI:** 10.1590/0100-3984.2021.0098

**Published:** 2022

**Authors:** Tarso Augusto Duenhas Accorsi, Karine De Amicis Lima, José Roberto de Oliveira Silva Filho, Renata Albaladejo Morbeck, Carlos Henrique Sartorato Pedrotti, Karen Francine Köhler, Fabio de Castro Jorge Racy, Eduardo Cordioli

**Affiliations:** 1 Telemedicine Department, Hospital Israelita Albert Einstein, São Paulo, SP, Brazil.; 2 Israeli Institute of Social Responsibility, Hospital Israelita Albert Einstein, São Paulo, SP, Brazil.

**Keywords:** Telemedicine, Point-of-care systems, Ultrasonography, Emergencies, Coronavirus infections, Telemedicina, Sistemas automatizados de assistência junto ao leito, Ultrassonografia, Emergências, Infecções por coronavírus

## Abstract

**Objective:**

To evaluate the feasibility of telemedicine using a standardized multiorgan ultrasound assessment protocol to guide untrained on-site general practitioners at a field hospital during a life-threatening crisis.

**Materials and Methods:**

We evaluated 11 inpatients with shock, with or without acute dyspnea, for whom general practitioners spontaneously requested remote evaluation by a specialist.

**Results:**

All of the general practitioners accepted the protocol and were able to position the transducer correctly, thus obtaining key images of the internal jugular vein, lungs, and inferior vena cava when guided remotely by a telemedicine physician, who interpreted all of the findings. However, only four (36%) of the on-site general practitioners obtained the appropriate key image of the heart in the left parasternal long-axis view, and only three (27%) received an immediate interpretation of an image from the remote physician. The mean evaluation time was 22.7 ± 12 min (range, 7-42 min).

**Conclusion:**

Even in life-threatening situations, untrained general practitioners may be correctly guided by telemedicine specialists to perform multiorgan point-of-care ultrasound in order to improve bedside diagnostic evaluation.

## Abstract (pt)

**Objetivo:**

Avaliar a viabilidade da orientação por telemedicina de médicos *in situ* não treinados na avaliação ultrassonográfica de múltiplos órgãos mediante protocolo padronizado, durante uma situação de risco de vida em hospital de campanha.

**Materiais e Métodos:**

Avaliamos 11 pacientes com choque e/ou dispneia de manifestação aguda durante a internação, cujos clínicos gerais solicitaram auxílio de especialista a distância.

**Resultados:**

Todos os médicos aceitaram o protocolo e, posicionando o transdutor, obtiveram imagens-chave da veia jugular interna, pulmão e veia cava inferior, quando guiados por um médico via telemedicina, que interpretou os achados desses órgãos. No entanto, apenas quatro (36%) médicos *in situ* obtiveram a imagem-chave apropriada do coração na janela paraesternal do eixo longo esquerdo e três (27%) tiveram imagem remotamente interpretada imediatamente. O tempo de avaliação variou de 7-42 minutos (média de 22,7 ± 12 minutos).

**Conclusão:**

Em situação de risco de vida, os clínicos gerais não treinados podem ser corretamente orientados por especialistas em telemedicina para realizar ultrassonografia multiórgãos *in situ*, melhorando o diagnóstico beira do leito.

## INTRODUCTION

Ultrasound is one of the most cost-effective and versatile medical imaging techniques. Sonographers have utilized it in prehospital emergency medicine, emergency departments, operating rooms, intensive care units, and outpatient clinics, as well as for the management of mass casualties and disasters. Lightweight portable ultrasound devices are widely available and have demonstrated effectiveness in improving diagnostic accuracy, even under field conditions^(1)^.

Remote ultrasound is already used in some situations in geographically inaccessible areas^(2)^. Remote prehospital ultrasound is feasible, mainly in trauma settings^(3)^. A meta-analysis of 28 studies of telesonography in emergency medicine confirmed the feasibility and high diagnostic accuracy of the technique, as well as its clinical and educational utility. Nevertheless, there are no precise data regarding protocols, acquisition time, or image interpretation for telesonography, nor regarding its relevance in changing medical practice^(4)^. Some case reports have shown that telesonography provides benefits in the standardized assessment of cardiac function in patients with shock^(5)^.

The coronavirus disease 2019 (COVID-19) pandemic resulted in many critical cases and a reorganization of the health care system, including the rapid construction of field hospitals with massive recruitment of general practitioners^(6)^. In addition to respiratory complications, COVID-19 can have multiple serious systemic manifestations and require specialized assessment, including sonography, which is often unavailable to specialists in field hospitals^(7)^. Telemedicine can meet those demands, and telesonography can improve the assessment of patients with life-threatening conditions^(8)^. However, to our knowledge, there have been no studies of remote guidance of untrained physicians in the ultrasound assessment of patients with life-threatening conditions at field hospitals. This issue is relevant because it can reduce costs by precluding the need to have radiologists on site, because it is time-consuming to train general practitioners in the proper use of ultrasound, and because it is important that ultrasound be handled by a clinical care team.

The aim of this study was to evaluate telemedicine guidance using a standardized multiorgan sonographic assessment protocol in untrained general practitioners during the COVID-19 pandemic at a field hospital, in terms of its feasibility (defined as the number of tasks performed correctly and the examination time). We hypothesized that telemedicine-guided point-of-care ultrasound (POCUS) would be feasible and effective in life-threatening situations, even when employed by untrained general practitioners. Our findings could lay the groundwork for future controlled studies.

## MATERIALS AND METHODS

### Population

This was a prospective descriptive study, involving a single telemedicine center (Hospital Israelita Albert Einstein) that was a reference for the Pacaembu COVID-19 Field Hospital, in the city of São Paulo, Brazil, and a medical team of general practitioners without any formal or informal training in the use of ultrasound. We included consecutive inpatients with COVID-19 who developed life-threatening complications and were being treated by local staff who spontaneously requested a real-time teleconsultation. There was no institutional protocol that mandated remote evaluation by a specialist, and the decision to request a teleconsultation was therefore made at the discretion of the on-site general practitioners. The study period was from March to June of 2020 (from the beginning to the end of the field hospital activities). The government-operated field hospital was designed to provide care for COVID-19 cases of low-to-moderate risk. Over the course of its operation, there were 1,515 admissions, 1,212 discharges, 289 high-complexity hospital referrals, and only three in-hospital deaths. We included the remaining inpatients, all of whom had shock, with or without acute dyspnea. After it had been confirmed that the audio and video were functioning correctly, the teleconsultation was performed in the presence of the patient and general practitioner. Teleconsultations in which there were connectivity problems were excluded. All telemedicine providers on duty at the Hospital Israelita Albert Einstein were senior emergency medicine physicians who were fully certified in Advanced Cardiology Life Support and other emergency skills necessary to provide emergency health care at the institution (which is accredited by the Joint Commission International), including emergency POCUS. After the initial evaluation and when a clinical opportunity presented itself, the remote physician applied the ultrasound protocol with the local team. The study protocol was approved by the local institutional ethics board (Registration no. 31489920.4.0000.0071) and was designated TelePOCUS. All of the data obtained can be accessed from the digital records of the institution. This study had no sources of funding. There were no changes to methods or outcome measures after the start of the study. Data were collected and stored confidentially by telemedicine physicians who were not involved in the face-to-face care provided by the local team and who ensured the confidentiality of the patient data. All patients provided written informed consent. There was no follow-up; patient participation ended when the teleconsultation was completed. The study ended when the field hospital ceased its operations. We sought to assess technical skills under remote guidance and not clinical outcomes.

### Standardized ultrasound protocol

**Figure 1:**
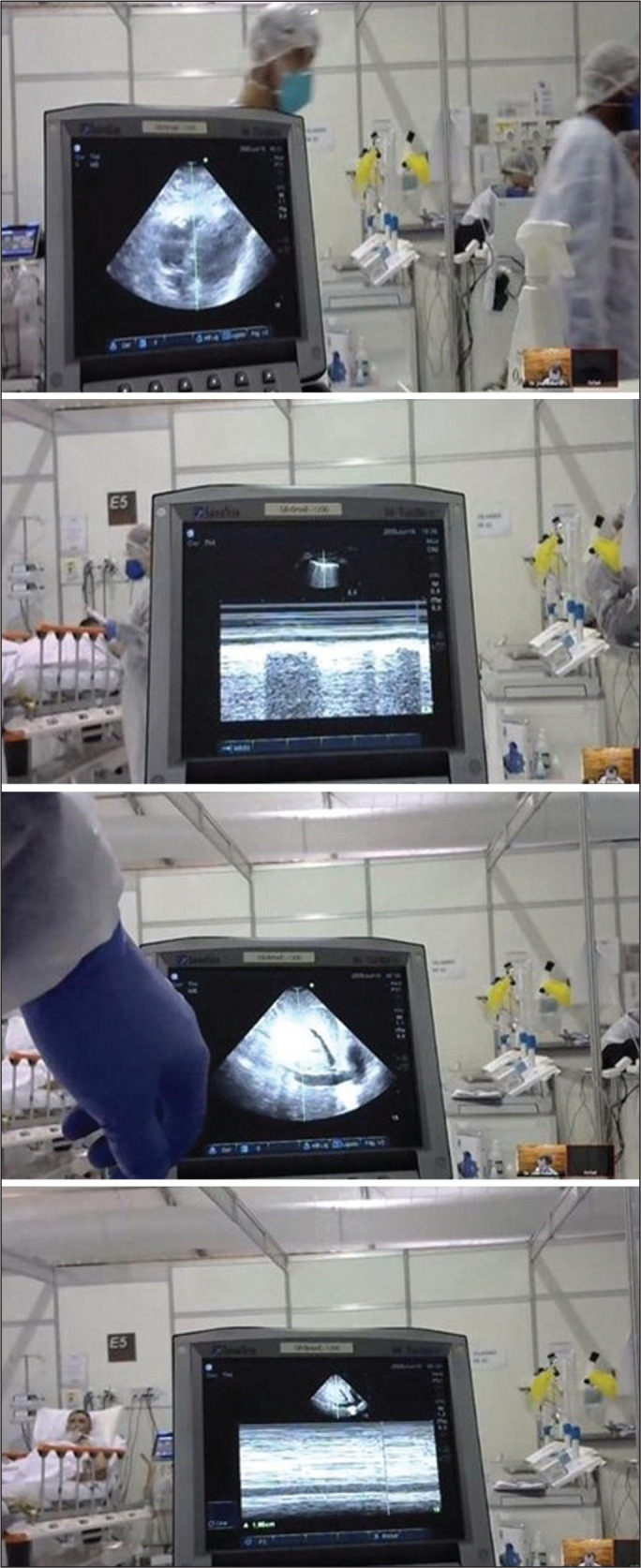
Screenshots of the telemedicine-guided bedside ultrasound performed at the field hospital.

**Figure 2:**
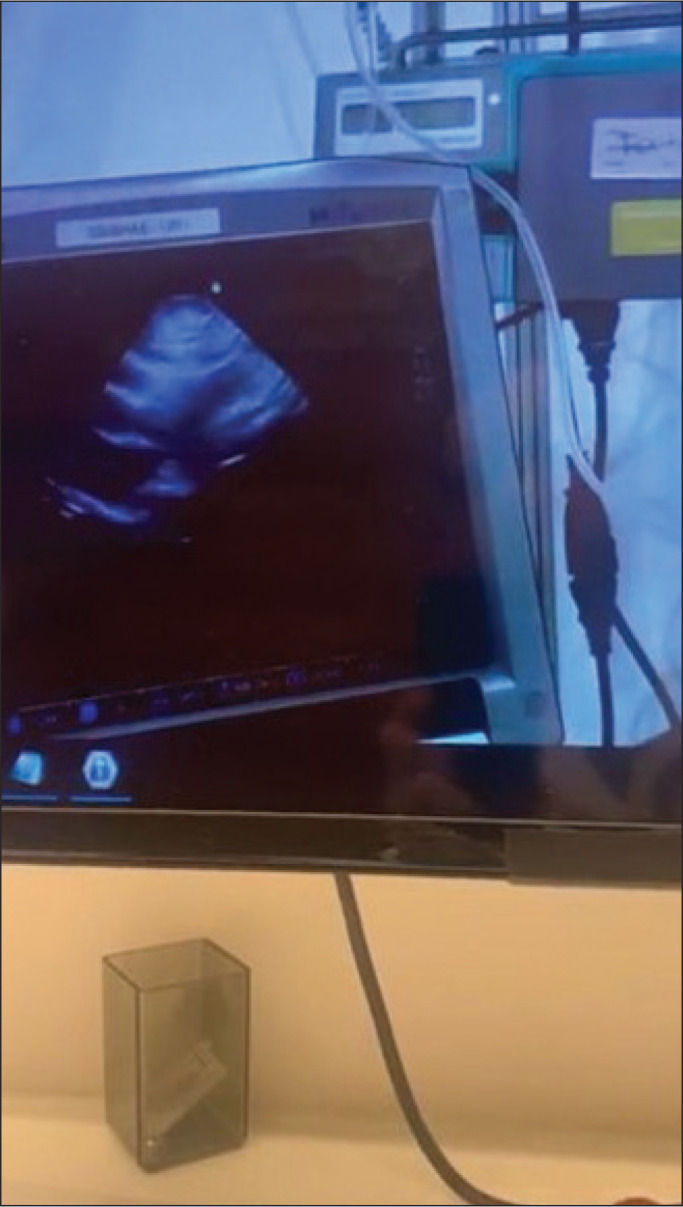
Image seen by the telemedicine physician.

All patients were evaluated with one of two lightweight portable ultrasound devices-Lumify (Koninklijke Philips N.V., Eindhoven, The Netherlands) or M-Turbo (Fujifilm Sonosite, Bothell, WA, USA)-with linear and convex transducers attached to a 10-in. tablet computer. The images were not transmitted to the telemedicine department and were evaluated remotely by the cardiologist on the screen of the tablet (Figures 1 and 2).

The proposed sonographic evaluation protocol aimed to analyze systolic dysfunction of the left ventricle, pulmonary interstitial-alveolar syndrome, systemic congestion, and possible fluid responsiveness. To that end, four areas were analyzed from top to bottom: the internal jugular vein (IJV); the heart; the lungs; and the inferior vena cava (IVC). The examination of each area was carried out in three steps: the transducer was positioned over the area of interest; the key image of the organ was obtained and the classical structures were identified; and any alterations were recognized. The IJV was the only area analyzed with the linear transducer, in a cross-sectional view of the supraclavicular region, and the absence of a change in diameter on exhalation was considered a predictor of right ventricular dysfunction and systemic congestion. The heart was analyzed only in the parasternal long-axis view, and systolic dysfunction was defined visually and subjectively (“eyeballing”). Each lung was scanned in four quadrants (anterosuperior, anteroinferior, superolateral, and inferolateral). Lung congestion was defined as at least three B-lines in two quadrants bilaterally. The IVC was analyzed in the subxiphoid region, and the suspicion of hypovolemia was raised by the collapse of the IVC during deep inspiration in non-intubated patients and by a > 50% decrease in its diameter during deep inspiration in intubated patients.

After remote guidance, the ability of the on-site physician to position the transducer properly in order to recognize the key image and to perform a dynamic interpretation of the images was categorized as “yes” or “no”. For each case, the total time to perform all three steps was counted from the beginning of the telemedicine call until the end of the ultrasound evaluation.

### Statistical analysis

All outcomes were summarized with the use of descriptive statistics. Continuous variables are described as means and standard deviations, whereas categorical variables are described as absolute and relative frequencies. Statistical analyses were performed with the IBM SPSS Statistics software package for Windows, version 22.0 (IBM Corp., Armonk, NY, USA).

## RESULTS

**Figure 3:**
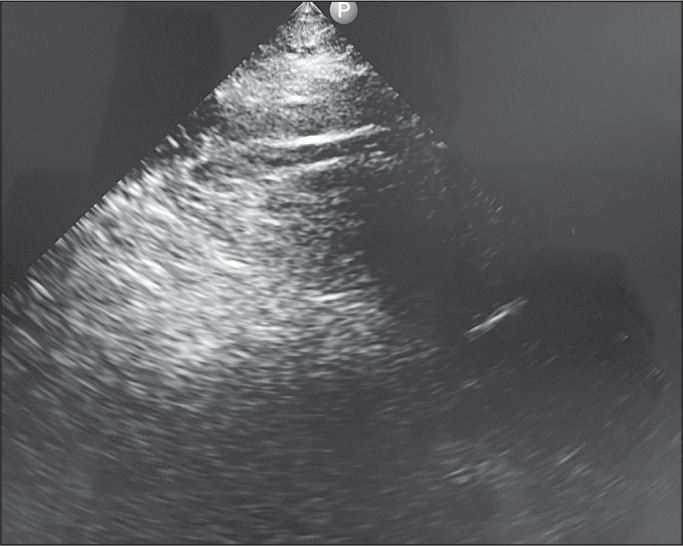
Set of three screenshots considered adequate for remote interpretation. A: Parasternal long-axis view of the heart. B: Single-quadrant view of a lung. C: IJV.

**Figure 4:**
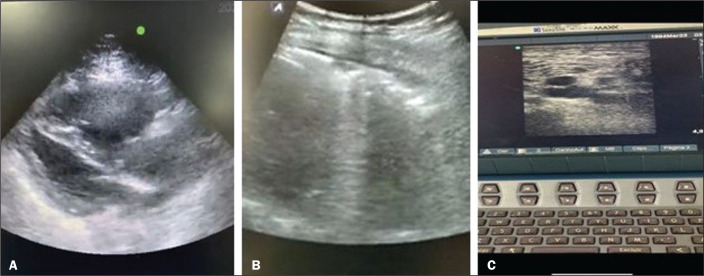
Screenshot of the heart that were considered inadequate for remote interpretation.

We evaluated the cases of 11 patients, which represented all spontaneous telemedicine requests during the operation of the field hospital under study. A total of eight untrained on-site general practitioners accepted the protocol and were able to position the transducer correctly, obtaining key images of the IJV, lung, and IVC under the guidance of a remote physician, who remotely interpreted all of the findings (Figure 3). One on-site general practitioner performed four evaluations, another performed two evaluations, and five performed one evaluation each. On the virtual side, seven telemedicine physicians provided guidance. Only four (36%) of the general practitioners obtained the appropriate key image of the heart in the left parasternal long-axis view, and only three (27%) obtained an image that was interpreted remotely in real time (Figure 4). As shown in Table 1, the mean total examination time was 22.7 ± 12 min (range, 7-42 min). Patients 1, 3, 10, and 11 were assessed by the same general practitioner; patients 2 and 5 were assessed by another general practitioner; and patients 4, 6, 7, 8, and 9 were each assessed by a different general practitioner. It is noteworthy that the general practitioner who assessed four patients did not have a clear progression in the total examination time, the first two examinations taking only 10 min each, whereas the last two took 16 min and 31 min, respectively. The same was true for the general practitioner who assessed two patients, in whom the examinations took 15 min and 36 min, respectively.

**Table 1:** Categorization of and time spent to complete the standardized telemedicine-guided POCUS protocol.

Patient	Proper transducer positioning	Key image acquisition				Image interpretation				Total time spent (min)
		IJV	Heart	Lung	IVC	IJV	Heart	Lung	IVC	
1	Yes	Yes	No	Yes	Yes	Yes	No	Yes	Yes	10
2	Yes	Yes	No	Yes	Yes	Yes	No	Yes	Yes	15
3	Yes	Yes	Yes	Yes	Yes	Yes	Yes	Yes	Yes	10
4	Yes	Yes	Yes	Yes	Yes	Yes	Yes	Yes	Yes	7
5	Yes	Yes	No	Yes	Yes	Yes	No	Yes	Yes	36
6	Yes	Yes	No	Yes	Yes	Yes	No	Yes	Yes	30
7	Yes	Yes	No	Yes	Yes	Yes	No	Yes	Yes	33
8	Yes	Yes	Yes	Yes	Yes	Yes	No	Yes	Yes	42
9	Yes	Yes	Yes	Yes	Yes	Yes	Yes	Yes	Yes	20
10	Yes	Yes	No	Yes	Yes	Yes	No	Yes	Yes	16
11	Yes	Yes	No	Yes	Yes	Yes	No	Yes	Yes	31
Mean	100%	100%	36%	100%	100%	100%	27%	100%	100%	22.7 ± 12

Although not the primary objective of the study, which had a technical focus, the effectiveness of POCUS in evaluating patients was analyzed, because it influenced the pre-test probability, resulting in a change in the diagnostic hypothesis in seven (63%) of the 11 cases. There was some overlap among the diagnoses: alveolar-interstitial syndrome was identified in seven cases; left ventricular systolic dysfunction was identified in three cases; systemic congestion was identified in two cases; and signs indicative of fluid responsiveness were observed in only one case.

## DISCUSSION

Telemedicine efficiently increases access to health care, particularly evaluation by specialists. Organized teleconsultation might mitigate the disease burden of conditions requiring emergency care^(9)^. Remote interpretation of cardiac POCUS handled by general practitioners and non-physician healthcare professionals has been shown to improve the diagnosis of cardiovascular disease in patients in low-resource areas^(10)^. Smith et al.^(11)^ showed that there is no difference in telemedicine accuracy among multiple fixed cameras, smartphones, and audio-only devices regarding the accuracy and feasibility of remote ultrasound guidance, from the perspective of an observer or from that of trainees. There is also evidence that remote teaching is as effective as in-person teaching for the acquisition of bedside ultrasound skills^(12)^. Levine et al.^(13)^ found that, via teleconsultation, intensivists were able to instruct non-physicians in the acquisition of ultrasound images of the right IJV, bilateral lung apices/bases, heart (subxiphoid view), and bladder, and that the quality of those images did not differ from that of images acquired directly by the intensivists.

Despite mounting evidence of the effectiveness of telemedicine guidance for the acquisition of ultrasound images, there have been few studies evaluating the form and timing of such guidance, especially of untrained professionals in life-threatening situations. In the present study, we evaluated the number of tasks performed correctly and the time required to complete the multiorgan POCUS protocol with remote guidance by a certified professional. The main feature was the spontaneous request for evaluation by a specialist in a life-threatening condition, characterized by the high emotional stress of an on-site team. It is also noteworthy that the majority of physicians staffing the emergency room were untrained generalists. We opted for a top-to-bottom evaluation because it is more didactic and easier to assimilate. Remote guidance achieved 100% effectiveness in the selection and positioning the transducer, as well as in the assessment of the IJV and IVC, regardless of the pre-test diagnosis and of whether the patient was intubated or not. In these evaluations, the telemedicine interpretation of the images was relatively simple.

In our sample, the major difficulty was the cardiac evaluation. It was decided that the inexperienced practitioners would not be instructed to evaluate the heart via the apical, four-chamber, or subxiphoid view, because of the difficulty in positioning the transducer and technical difficulties. In one-third of the patients, it was possible to interpret the parasternal long-axis image, although that was the most time-consuming part of the process. The decision to interrupt the cardiac evaluation was made by consensus among the physicians. That necessitated the analysis of other clinical data that could demonstrate ventricular dysfunction, although the time required to perform that analysis was not determined. In half of the cases in which it was not possible to evaluate the heart, the POCUS added value in the other multiorgan evaluations. Intubation hindered evaluation of the heart but not of the other organs. It is possible that the general practitioners who performed multiple screenings had a greater learning experience than did those who performed only one. Nevertheless, the proportion of screenings in which key images of the IJV, lungs, and IVC were obtained was 100% for all of the general practitioners. Even the two who performed multiple screenings had difficulty in obtaining key images of the heart: the one who performed two screenings failed in both attempts; and the one who performed four screenings succeeded in only one. The last successful assessment was made by one of the general practitioners who performed only one assessment. The number of assessments evaluated in our study was too small for any conclusions to be drawn about the skills acquired.

In addition to our finding that most of the steps evaluated are feasible, we found that the total time spent in the examination, although quite variable, was satisfactory, the prolonged time spent in the cardiac evaluation being notable. There was no increase in the speed of assessment by the two professionals who performed more than one screening. The subjective perception on the part of the on-site staff and telemedicine physicians was that the POCUS evaluation time did not hinder the care routine and added real value to the evaluation. The two general practitioners who performed more than one screening reported subjective improvement in a subsequent analysis. We found that the mean total time spent in assessing the four areas of interest was 22 min. It should be borne in mind that all of the patients had COVID-19, which required extensive contact precautions. In previous studies of life-threatening conditions^(14,15)^, the time of assessment has not been documented. In the present study, we did not address costs. However, the very low rate of general practitioner mismanagement of life-threatening situations in which ultrasound could facilitate the diagnosis demonstrates the low cost-effectiveness of having on-site radiologists. In addition, there is a low demand for remote evaluation, which therefore does not impede the usual routine of the telemedicine center, inferring that telemedicine is a rational use of resources. Our findings highlight the great ease of complementing the on-site clinical assessment with remotely guided ultrasound, even in life-threatening situations.

Our study has some limitations. The small sample size prevented us from performing a robust statistical evaluation. However, the sample was obtained prospectively after spontaneous specialist telemedicine requests based on a real life-threatening situations that general practitioners found difficult to manage. In addition, the ultrasound evaluation of the heart involved only one view because it was performed in an emergency setting. Furthermore, we did not evaluate clinical outcomes.

## CONCLUSION

In a life-threatening situation, untrained general practitioners may be correctly guided by telemedicine specialists to perform multiorgan POCUS in order to improve bedside diagnostic evaluation. Telemedicine-guided ultrasound is feasible and can be performed rapidly, without hindrance to the institutional routine, even in situations that required extreme contact precautions, such as when treating patients with COVID-19. Our findings underscore the fact that telemedicine is an easily accessible, presumably cost-effective tool for specialist support and a fundamental strategy for restructuring the health system, even after the current pandemic. Controlled studies are needed in order to evaluate clinical outcomes in telesonography.
